# Multiscale dimensions of county-level disparities in opioid use disorder rates among older Medicare beneficiaries

**DOI:** 10.3389/fpubh.2022.993507

**Published:** 2022-09-26

**Authors:** Tse-Chuan Yang, Carla Shoff, Seung-won Emily Choi, Feinuo Sun

**Affiliations:** ^1^Department of Sociology, University at Albany, State University of New York, Albany, NY, United States; ^2^Independent Consultant, Baltimore, MD, United States; ^3^Department of Sociology, Anthropology, and Social Work, Texas Tech University, Lubbock, TX, United States; ^4^Global Aging and Community Initiative, Mount Saint Vincent University, Halifax, NS, Canada

**Keywords:** opioid use disorder, multiscale geographically weighted regression, spatial heterogeneity, geographic disparity, county

## Abstract

**Background:**

Opioid use disorder (OUD) among older adults (age ≥ 65) is a growing yet underexplored public health concern and previous research has mainly assumed that the spatial process underlying geographic patterns of population health outcomes is constant across space. This study is among the first to apply a local modeling perspective to examine the geographic disparity in county-level OUD rates among older Medicare beneficiaries and the spatial non-stationarity in the relationships between determinants and OUD rates.

**Methods:**

Data are from a variety of national sources including the Centers for Medicare & Medicaid Services beneficiary-level data from 2020 aggregated to the county-level and county-equivalents, and the 2016–2020 American Community Survey (ACS) 5-year estimates for 3,108 contiguous US counties. We use multiscale geographically weighted regression to investigate three dimensions of spatial process, namely “level of influence” (the percentage of older Medicare beneficiaries affected by a certain determinant), “scalability” (the spatial process of a determinant as global, regional, or local), and “specificity” (the determinant that has the strongest association with the OUD rate).

**Results:**

The results indicate great spatial heterogeneity in the distribution of OUD rates. Beneficiaries' characteristics, including the average age, racial/ethnic composition, and the average hierarchical condition categories (HCC) score, play important roles in shaping OUD rates as they are identified as primary influencers (impacting more than 50% of the population) and the most dominant determinants in US counties. Moreover, the percentage of non-Hispanic white beneficiaries, average number of mental health conditions, and the average HCC score demonstrate spatial non-stationarity in their associations with the OUD rates, suggesting that these variables are more important in some counties than others.

**Conclusions:**

Our findings highlight the importance of a local perspective in addressing the geographic disparity in OUD rates among older adults. Interventions that aim to reduce OUD rates in US counties may adopt a place-based approach, which could consider the local needs and differential scales of spatial process.

## Introduction

Life expectancy in the United States (US) has lagged behind other developed countries (1, 2). Since 2014, the US has witnessed a slight decrease in life expectancy, a phenomenon that is not observed in any other countries (3) before the COVID-19 pandemic. It has been suggested that the opioid epidemic contributes to this public health concern (4, 5) and several scholars have investigated the determinants of opioid-related deaths in US counties (6–8). However, little attention has been paid to opioid-related outcomes among older adults (age ≥ 65), which increasingly contribute to the ongoing opioid epidemic (9). One study uses Medicare data between 2013 and 2018 and reported that the prevalence of opioid use disorder (OUD) among older adults has increased by more than 3-fold. Specifically, there were approximately 4.6 OUD cases per 1,000 beneficiaries in 2013 and this overall prevalence soared to 15.7 in 2018. The elevating trend is universal across all racial/ethnic, gender, and socioeconomic groups (10).

There are three major reasons why older adults are vulnerable to OUD. First, due to the aging process, older adults are more likely to suffer from physical pain and mental illness than younger populations (11, 12). As such, older adults are frequent recipients of prescription opioids to manage their health conditions. As exposure to prescription opioids is positively associated with the development of OUD (13), older adults are likely to develop a particularly high risk of OUD over time. Second, older adults' vulnerability to OUD, due to declining health conditions, may be further compounded by social and psychological risk factors associated with life course events (e.g., retirement and bereavement), such as social isolation, depression, and helplessness (9, 12, 14). These factors may aggravate the risk of OUD. Third, compared with younger adults, older adults are less likely to realize the negative consequences of opioid use (15) and are more likely to overlook OUD symptoms due to fear of substance-use stigma (11). Importantly, baby boomers (70–80 million people) are generally more tolerant or accepting of recreational substance use (11) than other generations, which likely makes baby boomers have lower perceived risk for not taking opioids as prescribed by their doctors.

Despite the unique vulnerability and challenges faced by older adults, little research has investigated the determinants of OUD until recently. Applying negative binomial regression to a 2017 county-level dataset, a study (16) finds that in a county, the number of older Medicare beneficiaries with OUD is associated with not only the beneficiaries' characteristics (e.g., average age), but also a county's socioeconomic conditions (e.g., social isolation). An individual-level study reports similar associations in that the risk of OUD is higher among socioeconomically marginalized older adults and those who reside in socially isolated and disadvantaged areas (17). Although these findings shed some light on the extant literature, the following gaps remain. First, the spatial distribution of OUD rates among older adults in US counties is unknown and the question of whether there is a geographic disparity in OUD rates across space has not been investigated. Second, previous research has suggested that spatial heterogeneity exists in county-level drug-overdose mortality and some scholars have explored this topic (7, 8). Nonetheless, it is unknown whether spatial heterogeneity is also embedded in the county-level patterns of OUD rates among older adults. Finally, most prior studies adopt a global modeling perspective to understand how OUD rates are shaped by other factors. This global modeling perspective assumes that the spatial process that leads to the observed ecological data is homogeneous but this assumption has been found to be unrealistic in empirical research (18). No prior research has applied a local modeling perspective to the research of OUD rates among older adults.

This study aims to fill these gaps by applying multiscale geographically weighted regression (MGWR) to a dataset of 3,108 contiguous US counties and county-equivalents and investigating three dimensions of spatial process, namely *level of influence, scalability*, and *specificity* (details in the next section). MGWR is a recently developed spatial analysis method that allows researchers to explore spatial non-stationarity (19, 20) and the three dimensions are drawn from the strengths of this local analysis perspective (21).

## Materials and methods

### Data sources and measures

This study assembles the analytical dataset from multiple national sources and focuses on the counties in the contiguous US (*N* = 3,108). The data from the Centers for Medicare & Medicaid Services (CMS) include beneficiary-level data from 2020 that is drawn from 3 CMS data files: (i) the Medicare Beneficiary Summary File (MBSF) Base segment, (ii) MBSF Chronic Conditions segment, and (iii) MBSF Other Chronic and Potentially Disabling Conditions Segment. The data have been limited to those beneficiaries who are 65 years of age or older and who are continuously enrolled in Medicare Fee-for-Services Parts A, B, and D for all 12 months of the 2020 calendar year and for all 12 months of 2019. Continuous enrollment for the previous data year is necessary due to the lookback period used to construct the OUD flag (discussed below). The beneficiary-level data are aggregated to the county-level based on county the beneficiary lives. The 2016–2020 American Community Survey (ACS) 5-year estimates (22) serve as the major source for the county-level socioeconomic features.

The dependent variable is the *OUD rate among older Medicare beneficiaries* (per 1,000 beneficiaries), which is defined as the total number of beneficiaries with OUD divided by the total number of beneficiaries in a county. OUD is defined using the overarching opioid use disorder flag that focuses on three opioid-related sub-indicators: (i) diagnosis and procedure code basis for OUD with at least one inpatient claim or two other non-drug claims of any service type with valid International Classification of Diseases, Tenth Revision (ICD-10) diagnosis codes or Current Procedural Terminology (CPT) or Healthcare Common Procedure Coding System (HCPCS) procedure codes, (ii) opioid-related hospitalization or Emergency Department visits, and (iii) use of medication assisted treatment (23). Furthermore, the following Medicare beneficiary characteristics are created at the county-level. *Percentage of female beneficiaries* is calculated by dividing the total number of female beneficiaries by the total number of beneficiaries. The *average age of beneficiaries* (in years) in a county is calculated. *Percentage of non-Hispanic white beneficiaries, percentage of non-Hispanic black beneficiaries*, and *percentage of Hispanic beneficiaries* are measured by dividing the number of beneficiaries in each racial/ethnic group by the total number of beneficiaries. Socioeconomically marginalized older adults may be eligible for both Medicaid and Medicare, which is known as dual-eligibility status. This study divides the total number of beneficiaries with dual-eligibility by the total number of beneficiaries to obtain the *percentage of dually eligible beneficiaries*.

The *average number of mental health conditions* is the mean value of beneficiaries' mental health conditions, including anxiety disorders, depressive disorders, bipolar disorder, and schizophrenia and other psychotic disorders. Consistent with defining OUD using the chronic condition and other chronic or potentially disabling condition flags, these mental health conditions are determined using the condition specific flags, which flag the beneficiary as having the condition during the calendar year if they meet the condition specific diagnosis or procedure code basis for that condition.^1^ Similarly, the *average number of physical conditions* refers to the mean value of beneficiaries' physical conditions including chronic obstructive pulmonary disease, diabetes, chronic kidney disease, and hypertension, ranging from 0 to 4. These physical conditions are also determined using the condition specific flags. The final beneficiary characteristic is the *average hierarchical condition category (HCC) score*. CMS develops an algorithm to calculate a beneficiary's potential Medicare cost. The HCC score is normalized to 1 and a beneficiary with a score that is <1 is less costly than a beneficiary with a score that is >1 (24).

With respect to socioeconomic features of a county, three composite variables are constructed with the ACS 5-year estimates. Principal component analysis (PCA) is first applied to the following four variables and the PCA score is used to gauge the *social isolation index* among older adults: percentage of older adults with a disability; percentage of older adults who were divorced, separated, or widowed; percentage of older adults having difficulty living independently; and percentage of older adults living in poverty. Each variable has a factor loading higher than 0.65 and more than 60 percent of the total variation can be explained by the first principal component. This social isolation index is designed by the United Health Foundation (25) and has been recently used in opioid-related research (17). Higher values indicate higher levels of social isolation among older adults in a county. In addition, following previous research (26), this study creates the *concentrated disadvantage index* by applying PCA to five variables: logged median family income; unemployment rate; percentage of families headed by women; percentage of the population age 25 and older without a high school degree; and percentage of households receiving public assistance (i.e., cash payments including Temporary Assistance to Needy Families and General Assistance). This measure of concentrated disadvantage focuses on the general population and higher PCA scores reflect stronger concentrated disadvantage. The factor loadings of the five variables are >0.55 and approximately 60 percent of the total variation can be explained by the first principal component. Finally, the average of two standardized variables: percentage of owner-occupied housing units and percentage of households living in the same housing unit for at least 5 years is used to measure *residential stability*. Higher values indicate higher levels of residential stability in a county.

### Statistical analysis

The multiscale geographically weighted regression (MGWR) (19) serves as the major analytic technique used in this study. As the MGWR is an extension of GWR (27), it is important to first introduce GWR and then discuss the strengths of MGWR. According to Fotheringham and colleagues (28), a general GWR can be expressed as below (27):


(1) $$\begin{array}{l} y_{i}\;=\;\overset{k}{\underset{j\;=\;1}{\sum }}\beta_{ij}x_{ij}+\varepsilon_{i}, \end{array}$$

Where *y*_*i*_ is the dependent variable for location (i.e., county in this study) *i*∈{1, 2, …, *n*}, *x*_*ij*_ refers to the *j*th independent variable (*j*∈{1, 2, …, *k*}) and β_*ij*_ is the estimated parameter (i.e., coefficient) for *x*_*ij*_. ε_*i*_ is the error term. The following matrix form can be used to calibrate the GWR coefficient at each location *i*:


(2) $$\begin{array}{l} {\hat{\text{β}}}_{\text{i}}\;=\;{\left({\text{X}}^{\text{T}}{\text{W}}_{\text{i}}\text{X}\right)}^{-1}{\text{X}}^{\text{T}}{\text{W}}_{\text{i}}\text{y},\;i\in \left\{1,\;2,\;\ldots ,n\right\}, \end{array}$$

Where **X** is the n^*^(k+*1)* matrix of independent variables (including the intercept), and **y** is the n^*^1 dependent variable vector, and **W**_**i**_ is the n^*^n spatial weighting matrix for a given location *i*. In **W**_**i**_, the spatial weights are obtained with a specific kernel function and a bandwidth. Under the GWR framework, the bandwidth is assumed to be the same across all independent variables. That is, the relationship between an independent variable and the dependent variable operates at the same spatial scale (i.e., bandwidth) (27) and the local estimates are calibrated with this assumption.

The constant spatial scale assumption may not be realistic for two reasons. On the one hand, in empirical research, some relationships do not vary by location but others are space-dependent (29). The former is known as the global relationship, whereas the latter refers to spatial heterogeneity. When both types of relationships exist in the observed data, the constant spatial scale assumption may misestimate the local coefficients. On the other hand, the differences in culture, social structures, norms, and values across space may lead the association between an independent and a dependent variable to operate at different spatial scales. For example, when observing the spatial correlation of a variable in a smaller spatial scale (e.g., counties within a state), researchers tend to identify similarities; however, when the spatial scale becomes larger (e.g., counties across multiple adjacent states), scholars are likely to find differences (18). As such, the constant spatial scale assumption may not fully reflect the spatial data generating process underlying the observed patterns.

MGWR aims to address these methodological issues by relaxing the constant spatial scale (i.e., bandwidth) assumption and allowing variable-specific optimized bandwidth (19, 30). This is the major difference between a MGWR and a GWR model. A MGWR model can be formulated as a generalized additive model (19):


(3) $$y\;=\;\sum {}_{j\;=\;1}^{k}f_{j}+\varepsilon$$

Where, **f**_**j**_ is a smooth function applied to the *j*th independent variable (31). Under the MGWR framework, each smooth function is a spatial parameter surface calculated with a bandwidth that is specific to the *j*th independent variable. MGWR calibrates estimates using a back-fitting algorithm (19). That is, compared with GWR, MGWR is more general and each independent variable has its own bandwidth, which forms a data generating process that allows not only global but also localized associations, which may operate at different spatial scales. It should be emphasized that MGWR standardizes all variables in the back-fitting algorithm, which facilitates the comparison of estimated coefficients across the unit of analysis. The adaptive bi-square kernel is used in this study to address the uneven spatial distribution of observations. Other technical details of MGWR can be found elsewhere (20, 28).

### Dimensions of geographic disparities

Yang and colleagues have recently exploited the strengths of MGWR to investigate three dimensions of spatial process and geographic disparities (21), namely *level of influence, scalability*, and *specificity*. Extending the three dimensions to this study, we define the level of influence as the percentage of older Medicare beneficiaries affected by a certain independent variable across the contiguous US counties. Based on the local estimates of an independent variable, we can first identify the counties where this independent variable is statistically significant and then sum the total number of beneficiaries in these counties. We then divide the sum by the total number of beneficiaries in the entire study area. If a variable is found to influence more than 50 percent of the entire population, this variable will be categorized into the primary influencer group; otherwise (i.e., ≤ 50 percent), it is a secondary influencer.

Regarding *scalability*, it can be defined with the calibrated bandwidth of a variable. Scalability has three groups: global, regional, and local. According to Yang et al. (21), when a calibrated bandwidth of a variable is >75 percent of the global bandwidth (i.e., the total number of counties in this study), it can be defined as a “global” factor. If the bandwidth of a variable is between 75 and 25 percent of the global bandwidth, it is regarded as a “regional” factor. When the bandwidth of a variable is smaller than 25 percent of the global bandwidth, this variable falls into the “local” factor group. While this interquartile range approach may be arbitrary, it has been used to detect spatial non-stationarity in GWR analysis (28).

The third dimension, *specificity*, takes advantage of the standardized coefficients yielded by MGWR. In this study, each county will have its own estimates of the independent variables and they can be compared *within* each county. Such a comparison helps researchers to identify the independent variable that has the strongest association (regardless of estimated direction) with the dependent variable. That is, an independent variable may demonstrate the strongest association in some counties but not in others. We will visualize the specificity dimension to show the uniqueness of a certain variable across space. It should be noted that in a conventional ordinary least squares (OLS) regression model, the magnitude of the standardized coefficient of an independent variable increases with the variance of this independent variable. This pattern may make coefficient comparisons problematic (32). Nonetheless, this concern cannot be directly applied to the MGWR framework because each variable has its own bandwidth and the comparison is within a county or the same population (21).

### Analytic strategy

This study conducts analysis in three phases: (I) conducting descriptive analysis and visualizing key variables, (II) implementing the OLS regression, which estimates the relationships between the independent variables and the dependent variable with data for all counties, and (III) using MGWR to obtain the local estimates (20). As MGWR generates abundant local parameter estimates, this study uses summary statistics and maps (33) to present the findings. Furthermore, the Monte Carlo method (20) is used to formally test whether spatial non-stationarity exists.

## Results

### Descriptive findings

Table 1 presents the descriptive statistics of the variables used in this study. On average, the OUD rate is 15.35 per 1,000 older Medicare beneficiaries in a county, which is comparable with the OUD rate reported in recent research (10). Regarding the county-level demographic composition of older Medicare beneficiaries, almost 60 percent of beneficiaries are female and the average age of beneficiaries is 75.83 years old. Slightly more than 88 percent of beneficiaries are non-Hispanic white, and non-Hispanic blacks and Hispanics account for 4.95 and 3.25 percent, respectively. Regarding dual-eligibility status, 15.85 percent of beneficiaries are eligible for both Medicare and Medicaid. The average numbers of mental conditions and physical conditions are 0.38 and 1.31, respectively. With respect to potential financial cost and health, the average HCC score is 1.08, indicating that the average financial burden at the county-level is greater than the population average. As the social isolation index and concentrated disadvantage index are created with PCA, they have a mean value of 0 with a standard deviation close to 1. Residential stability follows a similar pattern.

**Table 1:** Descriptive statistics of the variables used in this study (*N* = 3,108).

**Variable**	**Mean**	**SD**	**Min**	**Max**
Opioid use disorder (OUD) rate (per 1,000 beneficiaries)	15.35	10.05	0.00	148.22
Percentage of female (%)	58.21	2.37	46.08	73.33
Average age of beneficiaries (%)	75.83	0.73	72.07	79.47
Percentage of non-Hispanic (NH) white (%)	88.41	13.05	3.05	100.00
Percentage of non-Hispanic (NH) black (%)	4.95	9.36	0.00	75.17
Percentage of Hispanic (%)	3.25	8.97	0.00	96.85
Percentage of dual eligibility (%)	15.85	9.52	0.00	85.02
Average number of mental health conditions (count)	0.38	0.08	0.07	0.89
Average number of physical conditions (count)	1.31	0.22	0.50	2.08
Average hierarchical condition category (HCC) score (count)	1.08	0.12	0.61	1.83
Social isolation index	0.00	1.00	−3.24	4.94
Concentrated disadvantage index	−0.01	0.99	−2.42	6.96
Residential stability	0.01	0.88	−5.68	2.36

**Figure 1:**
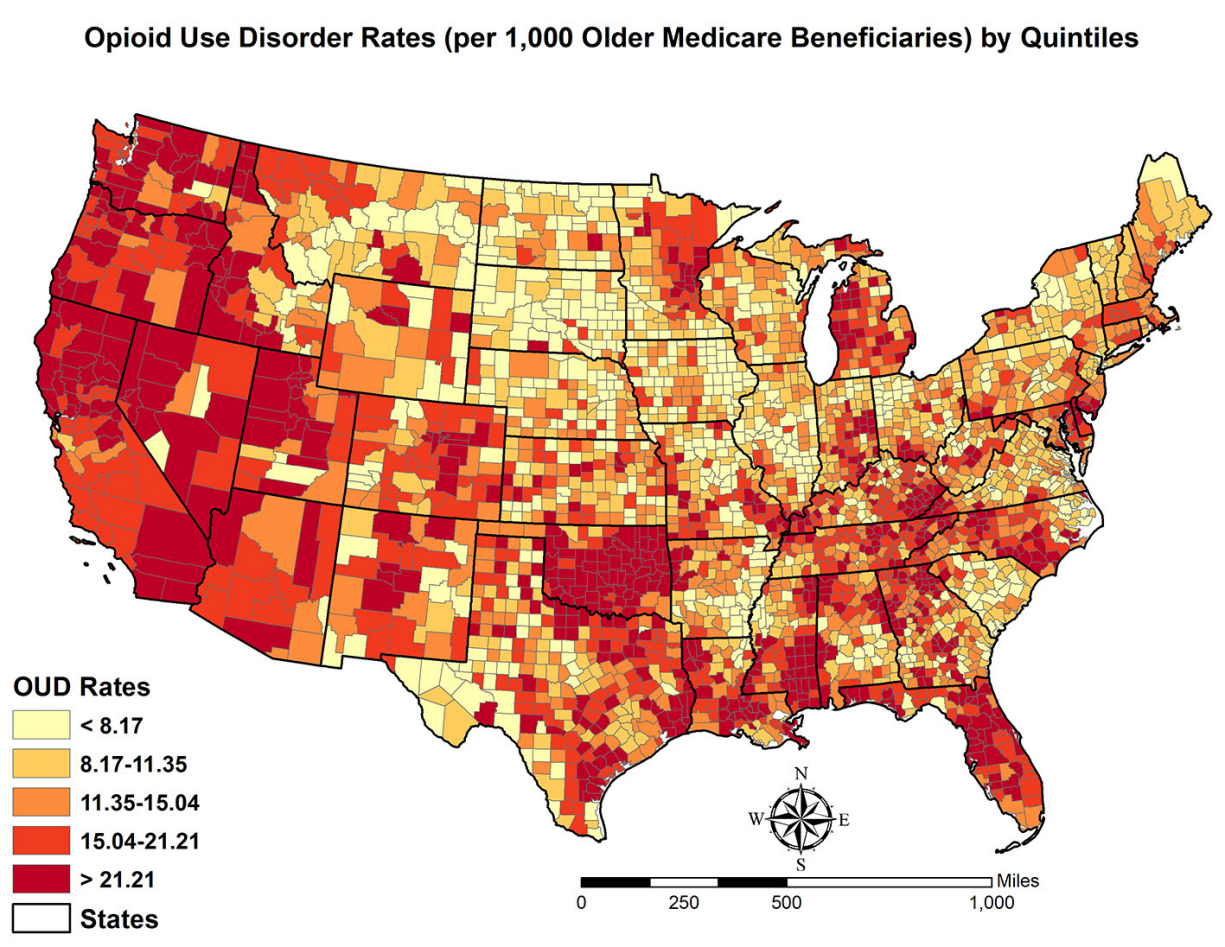
Spatial distribution of opioid use disorder rates (per 1,000 older Medicare beneficiaries) by quintiles in contiguous US.

The spatial distribution of OUD rates among older Medicare beneficiaries (by quintiles) is shown in Figure 1. Some patterns are notable. Counties with high OUD rates are mainly concentrated in the Pacific Coast and the Four Corners region. Counties in Oklahoma, Michigan, Mid-Appalachian Region, and along the Gulf of Mexico Coastal Region also report high OUD rates. By contrast, counties in Mid-West, Great Plains, and Northeastern states have low OUD rates. These patterns suggest that OUD rates are not evenly distributed across space. The spatial process that generates these patterns is likely to be place-dependent and spatial heterogeneity seems to exist in the data, which will be formally examined with the MGWR analysis.

### OLS and MGWR results

The OLS and MGWR results are summarized in Table 2. Specifically, columns (a) and (b) are drawn from the OLS analysis and columns (c) to (i) are based on the MGWR modeling. We discuss the main findings below. First, the global (OLS) estimates [i.e., column (a)] suggest that racial/ethnic composition, health conditions, and HCC score are associated with the OUD rate. For example, higher percentages of non-Hispanic black and Hispanic beneficiaries are associated with lower OUD rates at the county-level. Mental health conditions and HCC scores are positively related to OUD rates. It should be noted that the association between mental health conditions and OUD is opposite to that between physical health conditions and OUD. One plausible explanation is that the physical health conditions included in our measures are not strongly associated with pain but they require regular doctor visits, which may increase the awareness of opioid misuse or abuse. Beyond beneficiary characteristics, the OUD rate of a county increases with social isolation and decreases with residential stability. There is no significant relationship between concentrated disadvantages and the rate of OUD. Column (b) includes the variance inflation factors (VIF) among the independent variables. As all VIFs are smaller than 10 (the commonly used criterion), multicollinearity is unlikely to bias the estimates of standard errors of coefficients.

**Table 2:** OLS and MGWR results of opioid use disorder (OUD) rate (per 1,000 older medicare beneficiaries).

	**Global estimates (a)**	**VIF^†^ (b)**	**Mean (c)**	**SD (d)**	**Min (e)**	**Median (f)**	**Max (g)**	**Monte Carlo *p*-value (h)**	**MGWR Bandwidth (i)^‡^**
Percentage of female	−0.04	1.74	0.03	0.00	0.03	0.03	0.04	0.87	3,106
Average age of beneficiaries	−0.21***	1.50	−0.12	0.02	−0.16	−0.13	−0.09	0.12	2,359
Percentage of NH white	−0.07	9.95	−0.30	0.16	−0.56	−0.34	0.07	0.01	358
Percentage of NH black	−0.17***	5.24	−0.34	0.00	−0.34	−0.34	−0.33	0.94	3,106
Percentage of Hispanic	−0.09*	5.09	−0.26	0.10	−0.53	−0.24	−0.13	0.05	1,474
Percentage of dual eligibility	0.00	2.95	−0.05	0.00	−0.06	−0.05	−0.05	0.98	3,106
Average number of mental health conditions	0.11***	2.79	0.07	0.09	−0.09	0.07	0.31	0.02	384
Average number of physical conditions	−0.11**	4.78	−0.05	0.00	−0.06	−0.05	−0.05	0.57	3,106
Average HCC score	0.34***	4.95	0.41	0.36	−0.27	0.34	2.86	<0.001	44
Social isolation index	0.12***	2.49	−0.01	0.00	−0.01	−0.01	0.00	0.79	3,106
Concentrated disadvantage index	0.01	3.35	0.00	0.00	−0.01	−0.01	0.01	0.66	3,106
Residential stability	−0.05**	1.32	0.01	0.00	0.01	0.01	0.02	0.95	3,106
Intercept	0.00	–	−0.04	0.50	−0.98	−0.13	2.27	<0.001	44
AIC_C_	8,262.74		6,352.86						
Adjusted *R*^2^	0.17		0.61						

Significance: ^*^p <0.05, ^**^p <0.01, ^***^p <0.001.

^†^The variance inflation factors (VIF) among the independent variables are all smaller than 10, indicating that multicollinearity is not a concern.

^‡^The bandwidth is determined with the number of nearest neighbors for each location. This is a conventional approach in MGWR.

OLS, ordinary least squares; MGWR, multiscale geographically weighted regression; AICc, corrected Akaike Information Criterion.

Second, columns (c) to (g) are the summary statistics of the MGWR local estimates. Some variables are estimated to have divergent associations with OUD rates, while others show homogeneous relationships across space. Take the percentage of non-Hispanic white beneficiaries for example, its minimal local estimate [column (e)] is−0.56 but the maximal local estimate [column (g)] is 0.07. By contrast, the local estimates of the percentage of non-Hispanic black beneficiaries range between −0.34 and −0.33, suggesting a highly homogeneous relationship in US counties. The Monte Carlo test for spatial non-stationarity [column (h)] largely echoes the distribution of the local estimates for each variable. Three variables are found to have spatially varying associations with OUD rates, namely the percentage of non-Hispanic white beneficiaries, average number of mental health conditions, and average HCC scores.^2^ The three variables also have relatively small bandwidths compared with other covariates. The bandwidth of the percentage of non-Hispanic white beneficiaries is 358, which is comparable with that of the average number of mental health conditions (bandwidth = 384). The average HCC score has the smallest bandwidth of 44.

Third, in terms of model diagnosis, the corrected Akaike Information Criterion (AICc) is much smaller in the MGWR model (6,352.86) than the OLS model (8,262.74), indicating that the MGWR model is preferred and fits the data better.

### Spatial non-stationarity in OUD patterns

**Figure 2:**
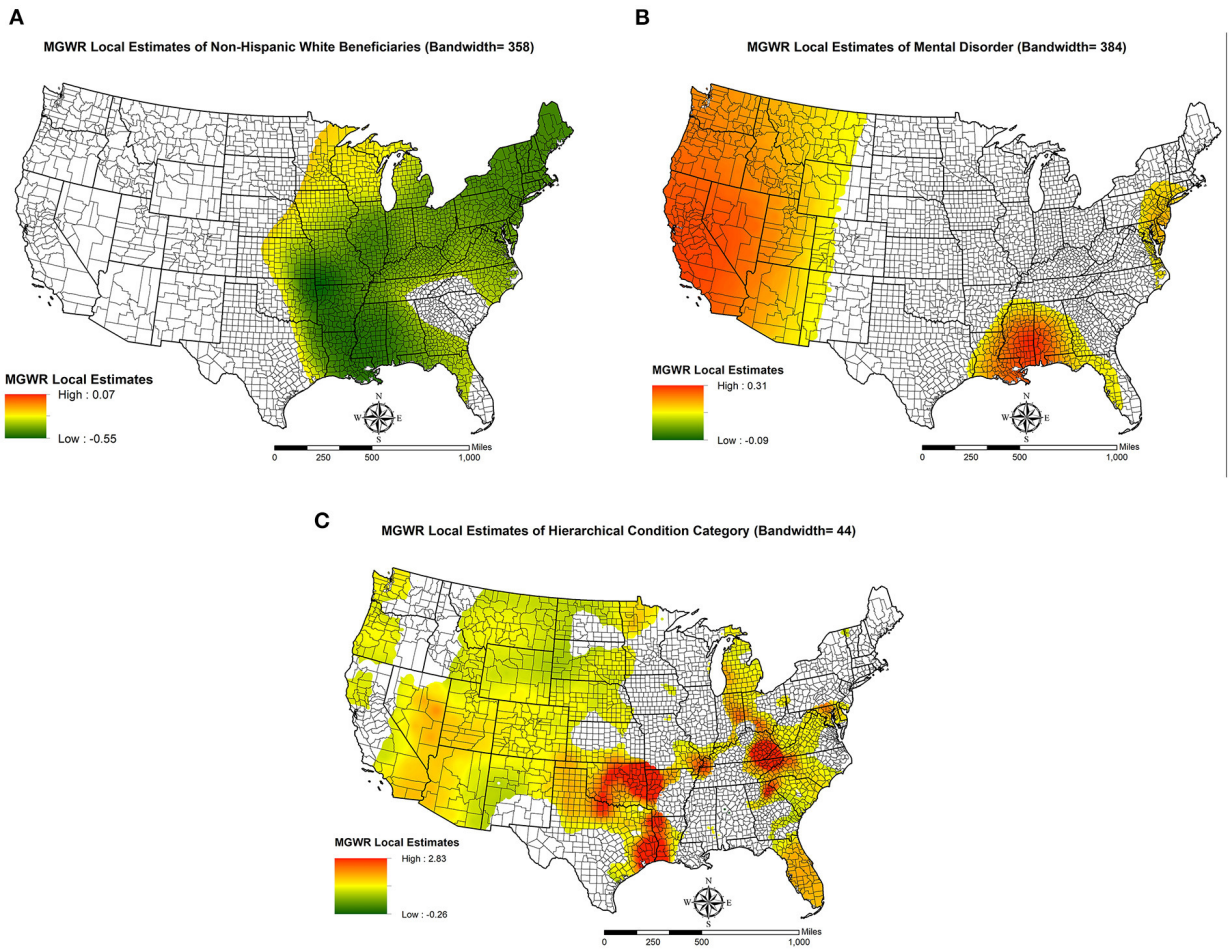
Spatial non-stationarity in the relationships between key independent variables and opioid use disorder rates (per 1,000 older Medicare beneficiaries) in US counties. **(A)** MGWR Local Estimates of Non-Hispanic White Beneficiaries (Bandwidth = 358); **(B)** MGWR Local Estimates of Mental Disorder (Bandwidth = 384); **(C)** MGWR Local Estimates of Hierarchical Condition Category (Bandwidth = 44).

**Figure 3:**
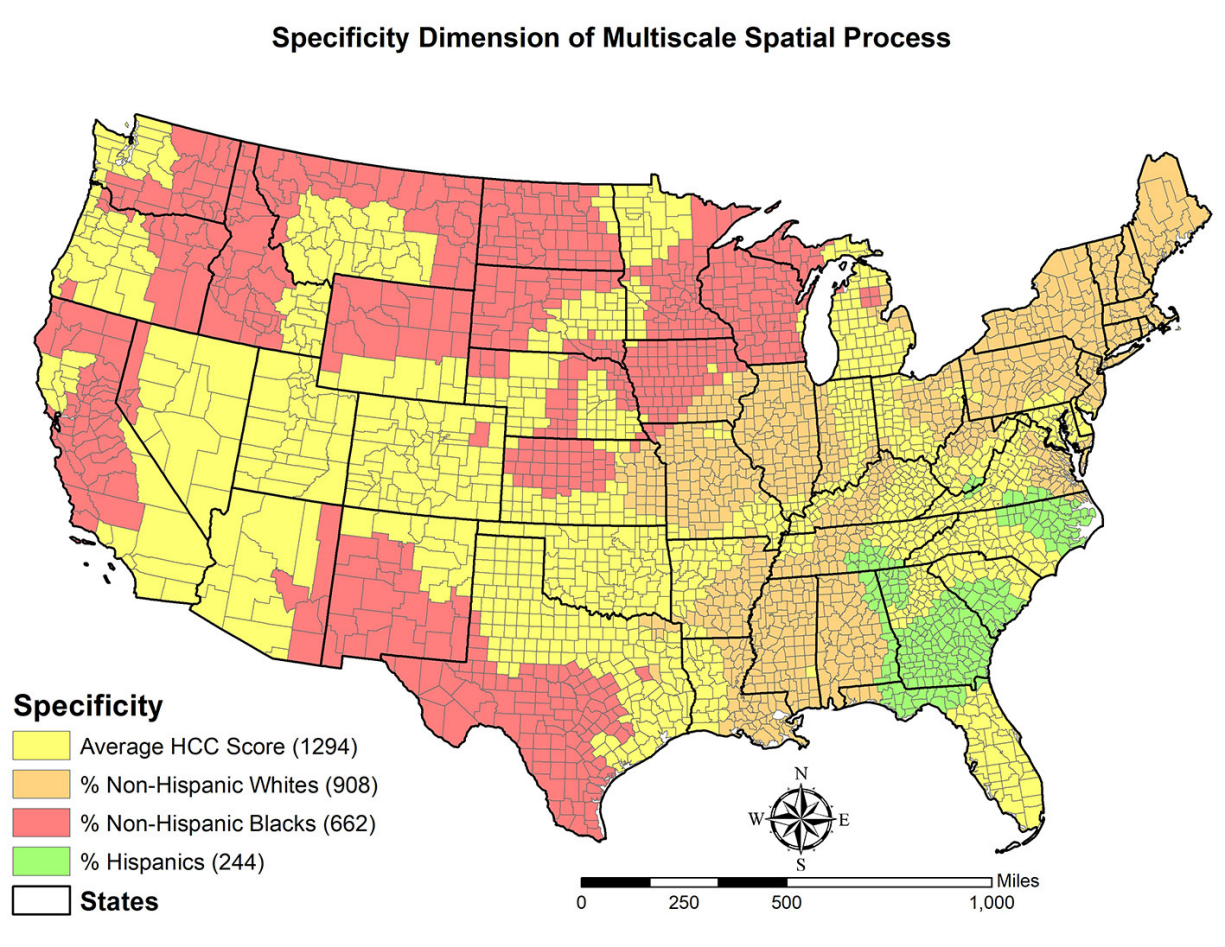
Specificity dimension of multiscale spatial process.

**Figure 4:**
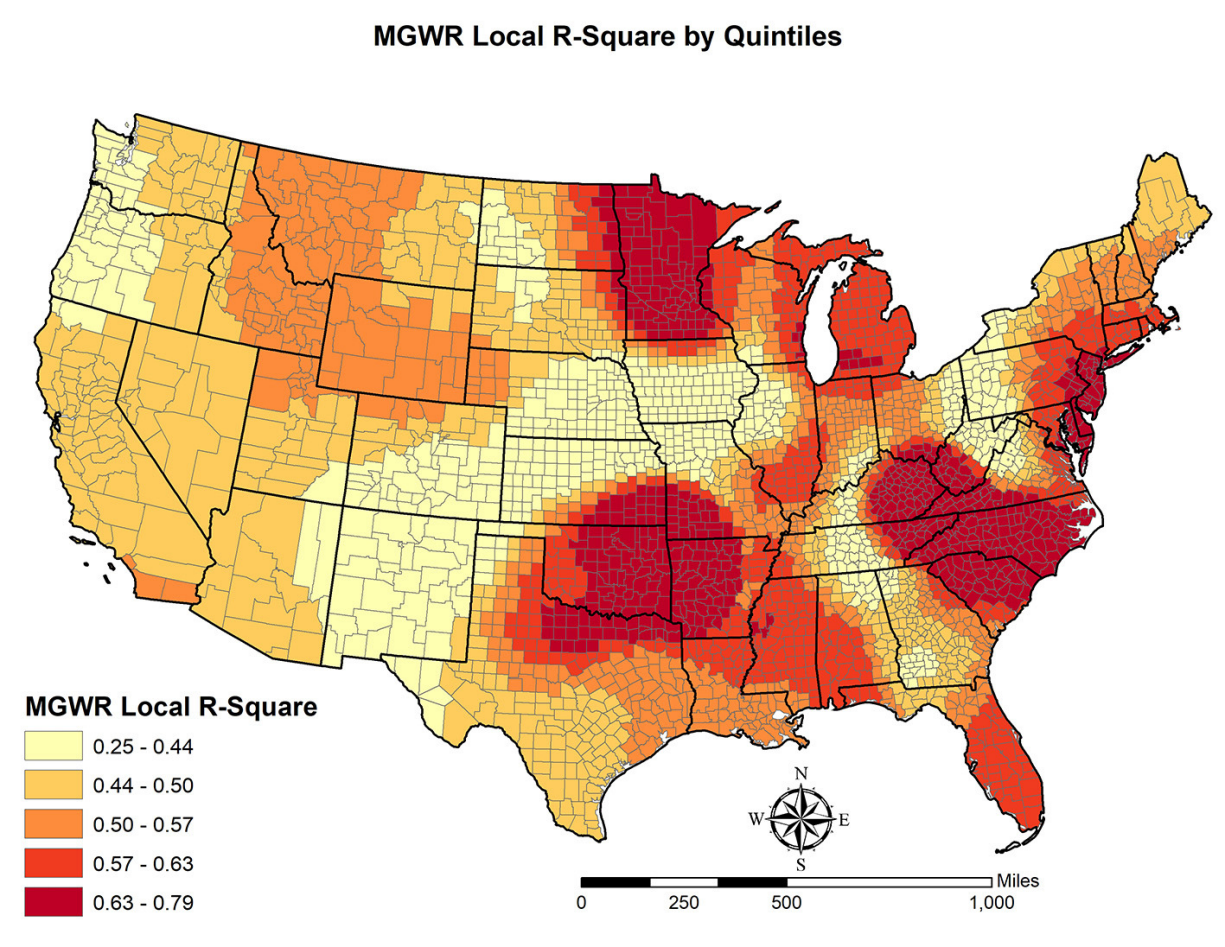
Local R-squares based on the multiscale geographically weighted regression.

To further demonstrate spatial non-stationarity, we visualize the MGWR results for the percentage of non-Hispanic white beneficiaries, average of mental health conditions, and average HCC scores in Figures 2–**4**. Before discussing the spatially varying associations with OUD, it should be noted that MGWR estimates are not statistically significant (*p*-value > 0.05) in white areas. Only colored areas refer to statistically significant associations. This visualization method has been commonly used in the geographically weighted regression literature (33).

Map A in Figure 2 shows the spatially varying relationship between the percentage of non-Hispanic white beneficiaries and OUD rates. The significant associations are found mainly to the east of Mississippi River with some exceptions including counties in South Carolina, northern Georgia, and southern Florida. The local associations suggest that higher percentages of non-Hispanic white beneficiaries are associated with lower OUD rates in these areas. We note that in the OLS estimates [Table 2, column (a)], the percentage of non-Hispanic white beneficiaries is not statistically significant. A plausible explanation for this discrepancy is that the positive local estimates offset the negative estimates, which leads to a null global relationship.

Map B in Figure 2 demonstrates how the average number of mental health conditions is related to OUD rates across space. The positive associations between these two variables are clustered in the West of US, particularly in the Pacific Coastal Region and Mountain States. The other two significant clusters are found in Louisiana, Alabama, Georgia, and New Jersey.

Map C in Figure 2 shows the spatial non-stationarity in the relationship between average HCC score and the OUD rate. There are two pockets where the local relationship is positively and strongly related to OUD rate (i.e., red areas). One is around Oklahoma, Eastern Texas and Western Louisiana. And the other is in Mid-Appalachian Regions, especially at the intersection between Virginia and Kentucky. Moreover, most counties in the Mountain States and Pacific Coastal Region are estimated to have a significant relationship.

### Multiscale dimensions of geographic disparities in OUD

The three dimensions of spatial process for each independent variable are presented in Table 3. Regarding the first dimension, *level of influence*, 5 variables are identified as primary influencers and 7 variables are secondary influencers. For example, the percentage of non-Hispanic black beneficiaries is a significant factor for OUD rate in every county so that all beneficiaries are affected by this variable. As such, this covariate is identified as a primary influencer. In contrast, approximately 41 percent of beneficiaries live in counties where the average number of mental health conditions is a significant factor, which is <50 percent and makes this covariate a secondary influencer.

**Table 3:** Three Dimensions of Multiscale Spatial Process for Each Independent Variable Based on the MGWR Models.

**Variable (bandwidth)**	**Level of influence^a^**	**Scalability^b^**	**Specificity^c^**
Percentage of female (3,106)	Secondary (0.8%)	Global	0
Average age of beneficiaries (2,359)	Primary (100.0%)	Regional	0
Percentage of NH white (358)	Primary (65.5%)	Local	908 (29.2%)
Percentage of NH black (3,106)	Primary (100.0%)	Global	662 (21.3%)
Percentage of Hispanic (1,474)	Primary (100.0%)	Regional	244 (7.9%)
Percentage of dual eligibility (3,106)	Secondary (0.0%)	Global	0
Average number of mental health conditions (384)	Secondary (40.9%)	Local	0
Average number of physical conditions (3,106)	Secondary (0.0%)	Global	0
Average HCC score (44)	Primary (52.1%)	Local	1,294 (41.6%)
Social isolation index (3,106)	Secondary (0.0%)	Global	0
Concentrated disadvantage index (3,106)	Secondary (0.0%)	Global	0
Residential stability (3,106)	Secondary (0.0%)	Global	0

^a^If the variable affects more than 50% of the total population, it is a primary influencer; otherwise (i.e., ≤ 50%), it is a secondary influencer. The percentage of population affected by a factor is included in the parentheses.

^b^If the bandwidth of a variable is larger than 75% of the global bandwidth (i.e., 2,331), it is a global determinant; if the bandwidth is smaller than 25% of the global bandwidth (i.e., 777), it is a local determinant; if the bandwidth is between 75% and 25% of the global bandwidth, it is a regional determinant.

^c^The number and percentage of counties that the focal variable has the strongest significant impact on the dependent variable (i.e., the largest absolute value of the coefficients that are statistically significant).

MGWR, multiscale geographically weighted regression.

In terms of the second dimension, *scalability*, 7 independent variables (e.g., percentage of female beneficiaries) have a bandwidth >75 percent of the global bandwidth (3,108^*^0.75 = 2,331) and they are categorized into the “global scale” group. Three variables have a bandwidth <25 percent of the global bandwidth (3,108^*^0.25 = 777). For example, the estimated bandwidth for the average HCC score is 44, which indicates that the OUD rate of a focal county is shaped by the nearest 44 counties. As such, this variable is associated with OUD rate at the “local scale”. Two independent variables, namely percentage of Hispanic beneficiaries and average age, have a bandwidth between 2,331 and 777 and they are defined as variables that operate to affect OUD rates at the “regional scale”.

With respect to *specificity*, 4 variables are found to have the strongest associations with OUD rates in US counties. Among them, average HCC score is estimated to be the most dominant variable in 1,294 of the total 3,108 counties (i.e., 41.6 percent). The percentage of non-Hispanic white beneficiaries is the most dominant factor in 908 counties, which is higher than the percentage of non-Hispanic black beneficiaries (662 counties) and the percentage of Hispanic beneficiaries (244 counties). We visualize the specificity dimension in Figure 3 and observe the following patterns. The average HCC score is the most dominant variable in most counties of Mountain States, such as Utah and Colorado, as well as Oklahoma and Northern Texas. Regarding the percentage of non-Hispanic white beneficiaries, it is mainly clustered in the Northeastern Region, Alabama, Illinois, and Missouri. The percentage of non-Hispanic black beneficiaries is found to have the strongest association with the OUD rate in Northern Great Plains, Idaho, Wisconsin, and along the US-Mexico border. Finally, the percentage of Hispanic beneficiaries is concentrated on Southern Georgia and part of the Carolinas.

The specificity dimension further illustrates spatial non-stationarity embedded in the geographic disparities in OUD rates among older adults. Explicitly, the relationship between a certain factor and the OUD rate within a county is not homogeneous across space in that either the direction or the magnitude of this relationship varies by location. In other words, the same change in a covariate may invoke different changes in the OUD rate and the differential responses depends on where a county is located and its surrounding counties.

MGWR also offers the local R-square for each county, which is visualized in Figure 4. The spatial pattern of local R-squares suggest that the model specification of this study fits the observed OUD rates best in the Great Lakes Region, Atlantic Coastal Region, most of the Black Belt region, and Oklahoma and its surrounding states.

## Discussion

With the results above, we revisited the three gaps in the extant literature. First, little is known about how OUD rates among older adults are distributed in US counties and the potential geographic disparity in OUD rates has not been explored. This gap can be filled with the exploratory spatial data analysis results of this study. Specifically, based on the 2020 Medicare data, counties with high OUD rates are concentrated in the West of the US with some pockets scattered in Oklahoma, Mid-Appalachian Region, and Florida. By contrast, counties with low OUD rates are in the Mid-West. Such a pattern suggests that the distribution of OUD rates among older adults is uneven and it is likely that spatial heterogeneity exists in US counties. More specifically, OUD rates tend to be place-dependent and different spatial scales reflect different spatial associations. For example, counties in the state of Oklahoma demonstrate a strong spatial dependence (i.e., counties with high OUD rates are nearby) whereas counties in the Northeast Region (e.g., Pennsylvania, New Jersey, and New York) may reflect spatial heterogeneity (i.e., OUD rates vary within thin this region).

Situating this finding into the literature, prior ecological studies either investigate the patterns within a single state or region (34, 35) or explore substance or opioid abuse among the general population (36), rather than older adults. After reviewing 46 published articles, Marks and colleagues (37) conclude that geospatial analysis techniques are commonly used in research of opioid-related outcomes. Nonetheless, no study has adopted a local spatial perspective to investigate the existence of spatial heterogeneity in ecological data. To our knowledge, this study is among the first to present such a spatial pattern of and geographic disparity in OUD rates among older adults in the contiguous US.

Second, several county-level studies have suggested that drug-overdose mortality is spatially heterogenous in that some factors are more important in certain counties than others (7, 8), but whether this argument can be applied to OUD rates among older adults is unknown. Drawing from the MGWR results, we found that at the county-level, only three variables have spatially varying associations with OUD rates and others operate at the global or regional level. That is, we obtain evidence for spatial heterogeneity underlying the pattern of OUD rates, but such evidence only comes from beneficiaries' characteristics, i.e., percentage of non-Hispanic white beneficiaries, average number of mental health conditions, and average HCC score. These variables demonstrate unique patterns, which are visualized in Figure 2.

How do our findings related to spatial heterogeneity contribute to the literature? For one, without a local modeling perspective, our OLS findings largely echo a recent ecological study (16). For example, social isolation is negatively associated with OUD rates and residential stability decreases OUD rates. With the MGWR results, we can confirm that these two variables (i.e., social isolation and residential stability) have a universal relationship with OUD across space. Furthermore, the relationship between average number of mental health conditions and OUD is only significant in the West of US and part of the Black Belt. The average HCC score also demonstrates a strong spatial heterogeneity pattern. While some scholars have used typology analysis to investigate spatial heterogeneity in opioid-related health outcomes (38), this approach does not explore spatial heterogeneity for each independent variable.

Finally, this study challenges the commonly used global modeling perspective in the literature and identified three dimensions of the spatial process that generates the observed OUD rates. In terms of the level of influence, this study concludes that beneficiaries' characteristics play a larger role in shaping OUD rates than the socioeconomic conditions of a county because racial/ethnic composition of beneficiaries and the average of HCC score are categorized as primary influencers. Regarding scalability, the MGWR results support the argument that different independent variables may operate at different spatial scales to affect OUD rates. All three types of scalability, namely global, regional, and local, are found in this study. This finding is similar to a recent study (21) and indicates that it may not be appropriate to adopt the constant spatial scale assumption. The third dimension, specificity, shows that four factors are estimated to have the strongest association with OUD rates. Among them, the average HCC score is the most dominant in more than 40 percent of the total 3,108 counties, followed by the percentage of non-Hispanic white beneficiaries.

The three dimensions of spatial process take advantage of the strengths of MGWR and serve as an alternative to illustrate and visualize spatial heterogeneity. To our knowledge, the three dimensions have not been applied to ecological OUD studies and this study is the first to describe these dimensions specific to the 2020 OUD rates among older adults. Research on the opioid epidemic has paid attention to middle-aged populations and the opioid use behavior among older adults is often overlooked. As opioid prescription and regulation is less restricted in the US healthcare systems than in other developed countries (39), the spatial heterogeneity and spatial process found in this study may be unique to the study population.

While MGWR has overcome several limitations of GWR, such as multiple testing and developing a local inferential statistics framework (19, 30), it still has some shortcomings and our results should be interpreted with these caveats in mind. First, the multicollinearity among local estimates remains likely to be a concern, even though using different weight matrices in the back-fitting algorithm may minimize multicollinearity. Second, the current MGWR is developed for continuous outcomes that largely follow a normal distribution. When the dependent variable is highly skewed or sparse, the MGWR estimates may not be reliable. Finally, the global estimates cannot be decomposed into MGWR local estimates. As such, the global and local parameters do not have a clear relationship.

This study is subject to several limitations. First, using a different geographic unit (e.g., states) may lead to different findings and conclusions, which is known as the modifiable areal unit problem (40, 41). While the Medicare data can be aggregated to ZIP codes, which is the most granular unit available at CMS, we opted not to use this unit to avoid the small area estimation problem (i.e., few beneficiaries in a ZIP code). Second, starting January 1, 2020, the Substance Use-Disorder Prevention that Promotes Opioid Recovery and Treatment (SUPPORT) for Patients and Communities Act was enacted. Under the SUPPORT Act, CMS is allowed to pay Opioid Treatment Programs through bundled payments for OUD treatment services including FDA approved medications for OUD and related services (e.g., substance use counseling and periodic assessments). As such, we may observe more beneficiaries with OUD than previous years and analyzing data before 2020 may yield different results. Third, the cross-sectional research design does not allow us to make any causal inference and the findings cannot be generalized to other age populations.

Several policy implications can be drawn from this study. One is that the one-model-fits-all or global approach may not effectively address the increasing OUD rates among older adults. The MGWR findings suggest that policies aiming to lower OUD rates should focus on counties with high average HCC scores and high percentages of non-Hispanic white beneficiaries (the top two variables in the specificity dimension). In addition, higher average age of beneficiaries is also an important factor as this variable is significant in all counties. It may be necessary to prioritize resources to counties with higher concentrations of beneficiaries in the middle-old (ages 75–84) and the old-old (85+) age ranges. Finally, more attention should be paid to the place-based policies so that the differences in culture, values, attitudes, norms, and socioeconomic conditions across space can be explicitly considered in possible interventions.

## Data availability statement

The data analyzed in this study is subject to the following licenses/restrictions: The beneficiary level data can be accessed through the Research Data Center. Requests to access these datasets should be directed to Research Data Center, resdac@umn.edu.

## Author contributions

T-CY conceptualized this study, led the writing of the manuscript, and conducted the major analysis. CS managed the county-level data and contributed to the writing of the manuscript. S-wC interpreted findings, participated in writing, and critically commented on the manuscript. FS visualized, interpreted findings, and participated in writing. All authors read and approved the final manuscript.

## Funding

T-CY received the Interdisciplinary Research Network on Rural Population Health and Aging, which is funded by the National Institute on Aging (R24-AG065159).

## Conflict of interest

The authors declare that the research was conducted in the absence of any commercial or financial relationships that could be construed as a potential conflict of interest.

## Publisher's note

All claims expressed in this article are solely those of the authors and do not necessarily represent those of their affiliated organizations, or those of the publisher, the editors and the reviewers. Any product that may be evaluated in this article, or claim that may be made by its manufacturer, is not guaranteed or endorsed by the publisher.

## Footnotes

^1^Information on how each of the chronic conditions and other chronic or potentially disabling conditions are defined can be found on the Chronic Conditions Data Warehouse website: https://www2.ccwdata.org/web/guest/condition-categories.

^2^The percentage of Hispanic beneficiaries is marginally significant (*p*-value = 0.052) so we exclude it from the discussion.
